# Supplementary material for: Multiomic characterization of pancreatic cancer-associated macrophage polarization reveals deregulated metabolic programs driven by the GM-CSF–PI3K pathway
Source: eLife. 2022 Feb 14;11:e73796. doi: 10.7554/eLife.73796 (PMC8843093; doi:10.7554/eLife.73796)
Supplement: Source data 1. [file elife-73796-data1.pptx]

## Slide 1
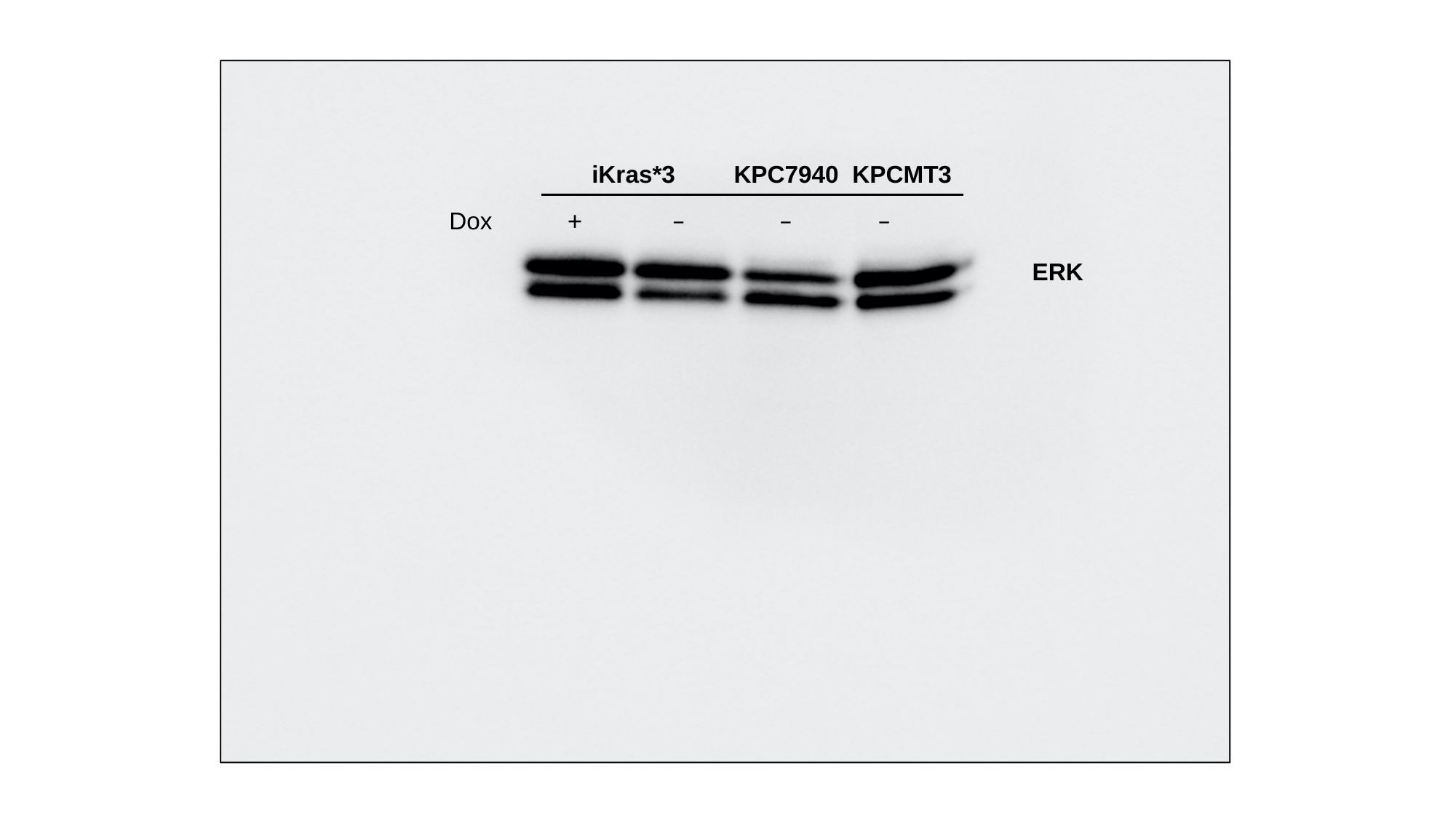

iKras*3
KPC7940
KPCMT3
+
–
–
–
Dox
ERK

## Slide 2
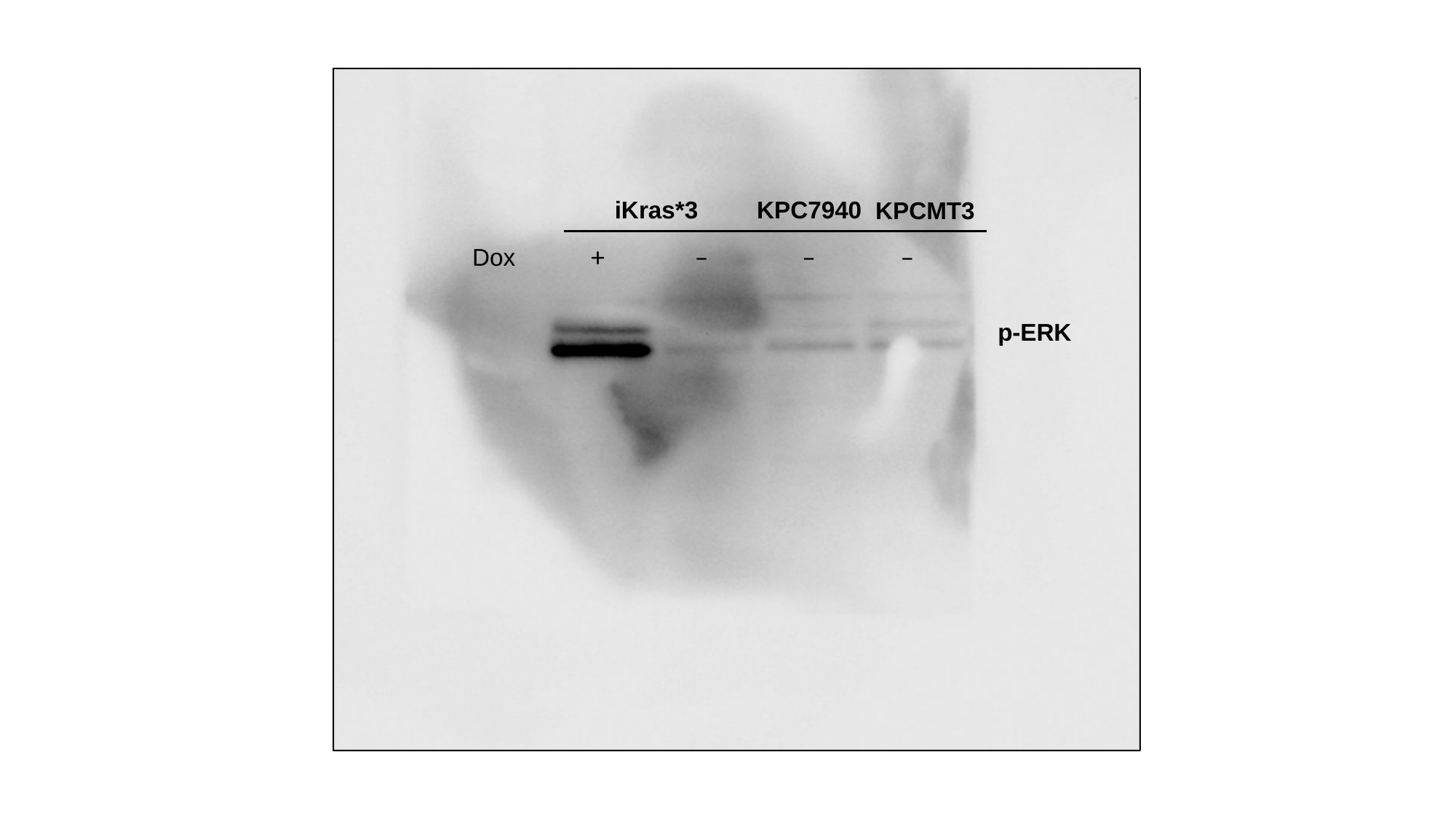

iKras*3
KPC7940
KPCMT3
+
–
–
–
Dox
p-ERK

## Slide 3
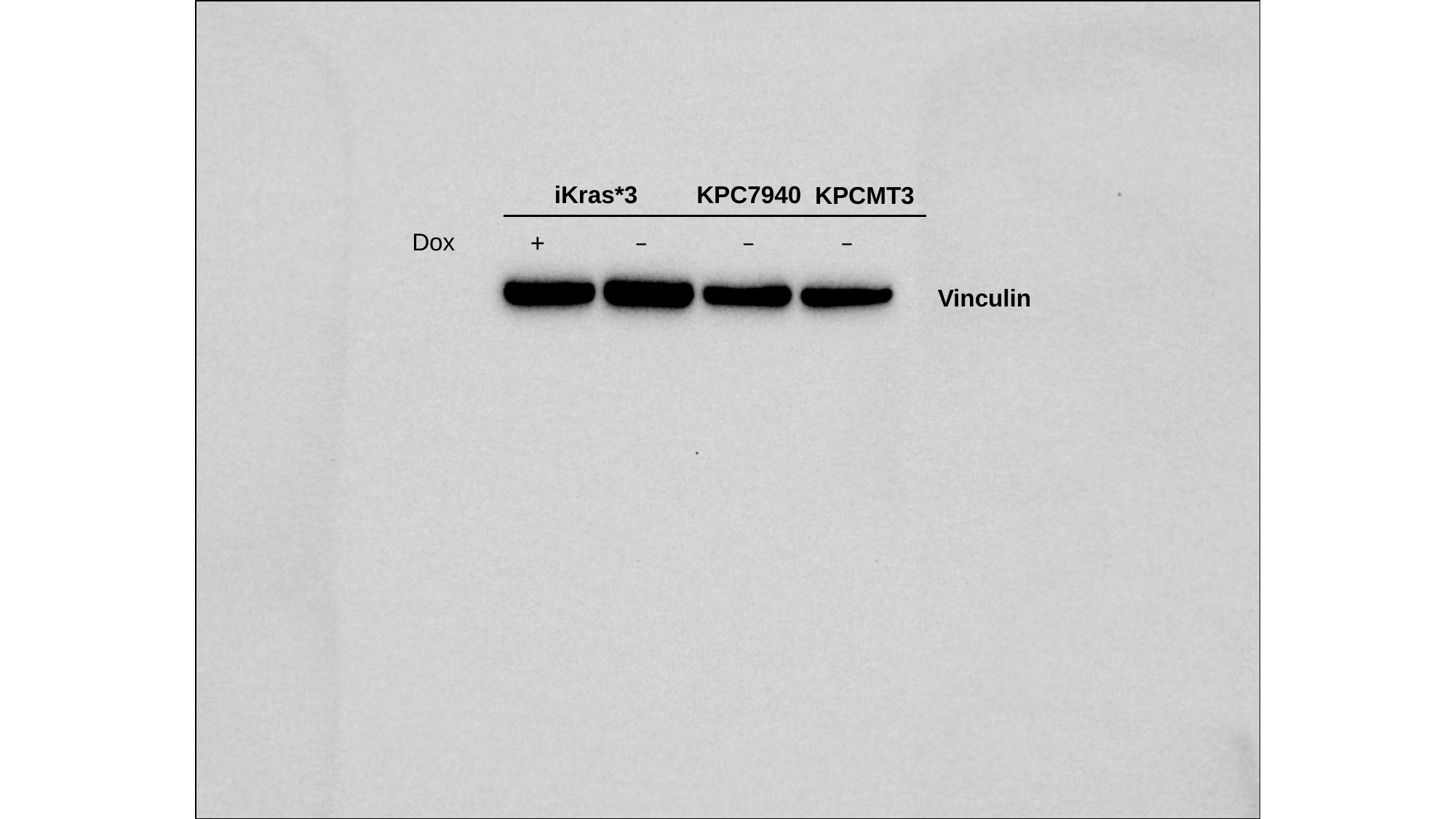

iKras*3
KPC7940
KPCMT3
+
–
–
–
Dox
Vinculin

## Slide 4
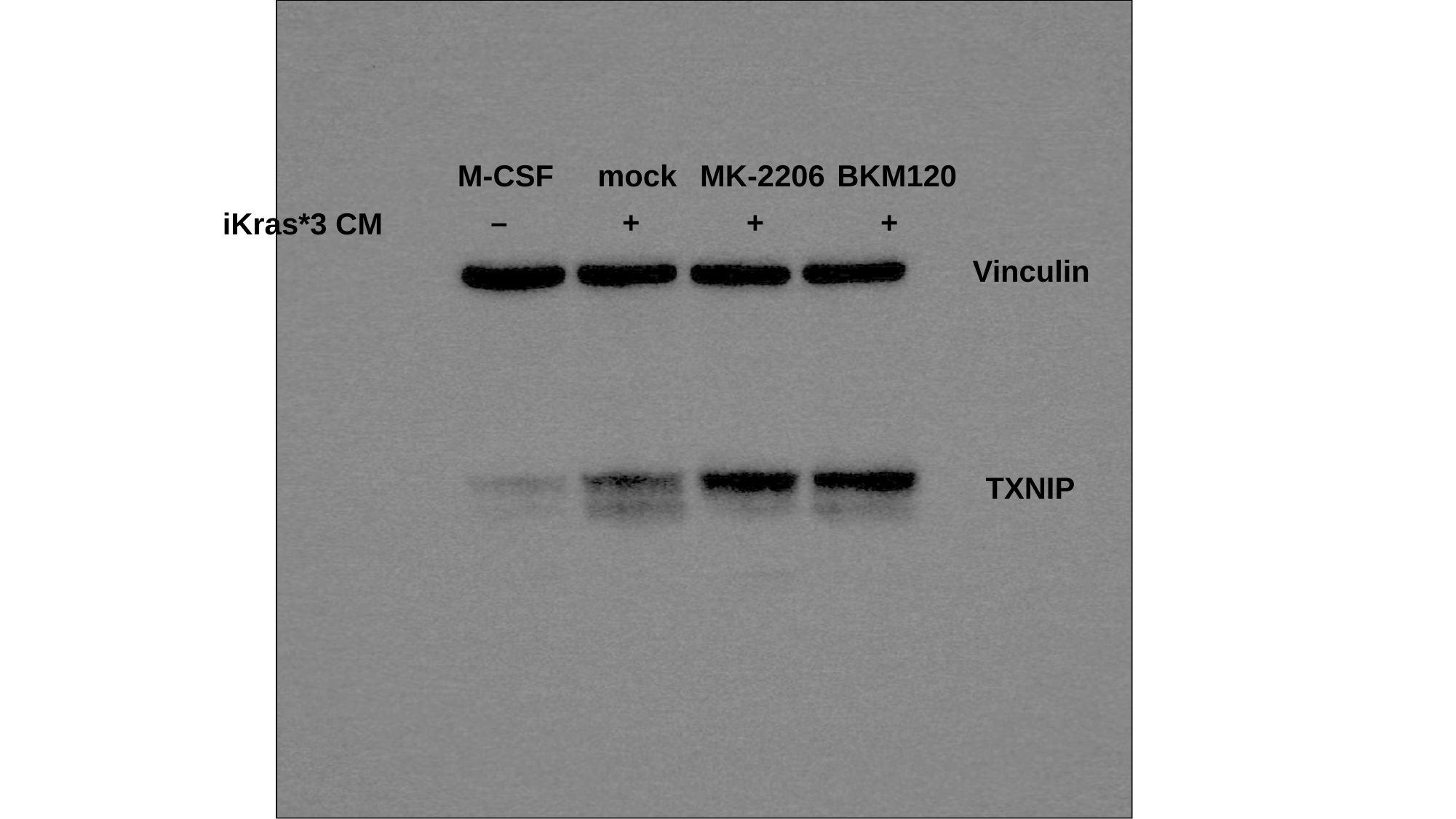

M-CSF
mock
MK-2206
BKM120
–
+
+
+
iKras*3 CM
Vinculin
TXNIP

## Slide 5
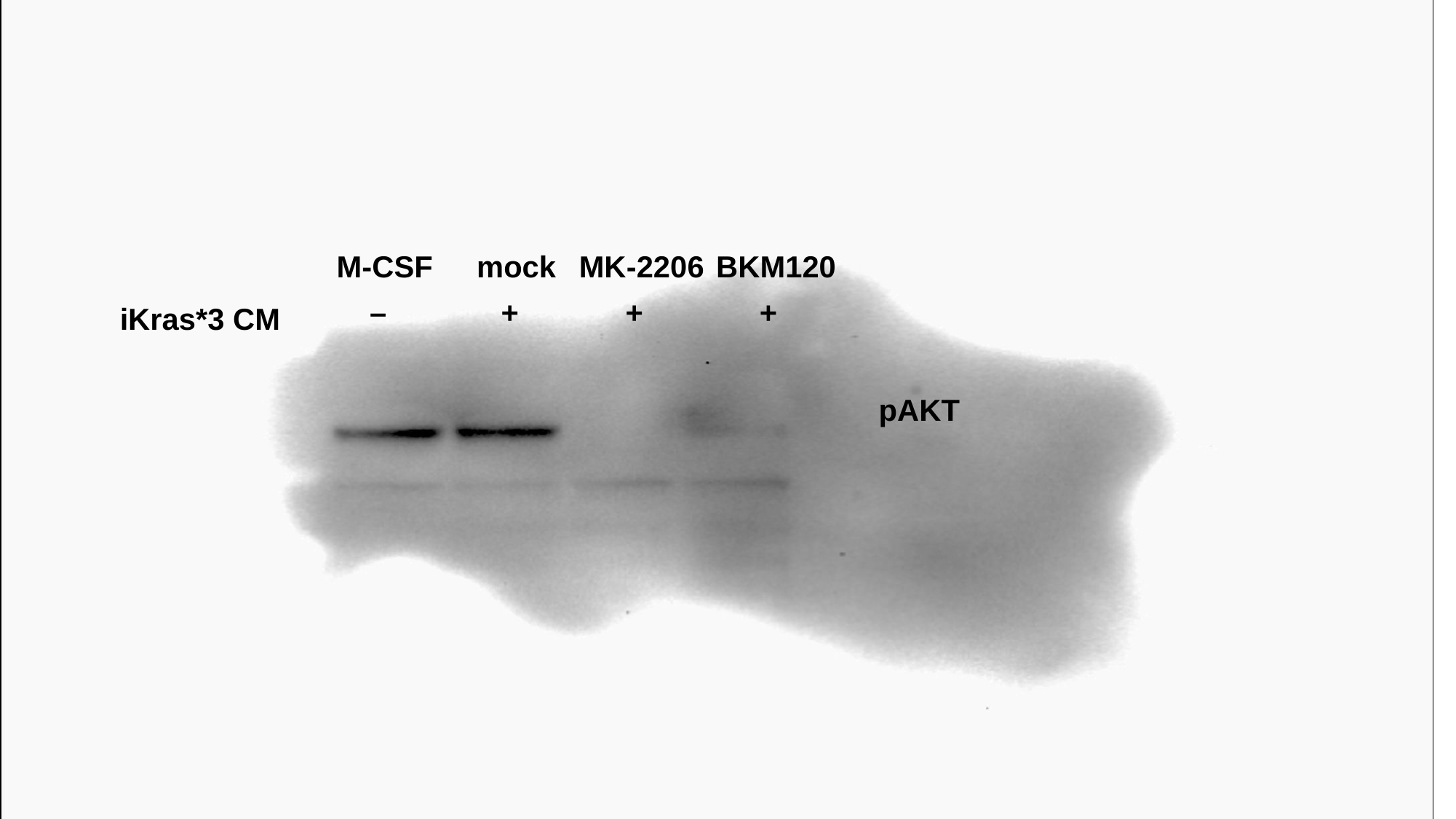

M-CSF
mock
MK-2206
BKM120
–
+
+
+
iKras*3 CM
pAKT

## Slide 6
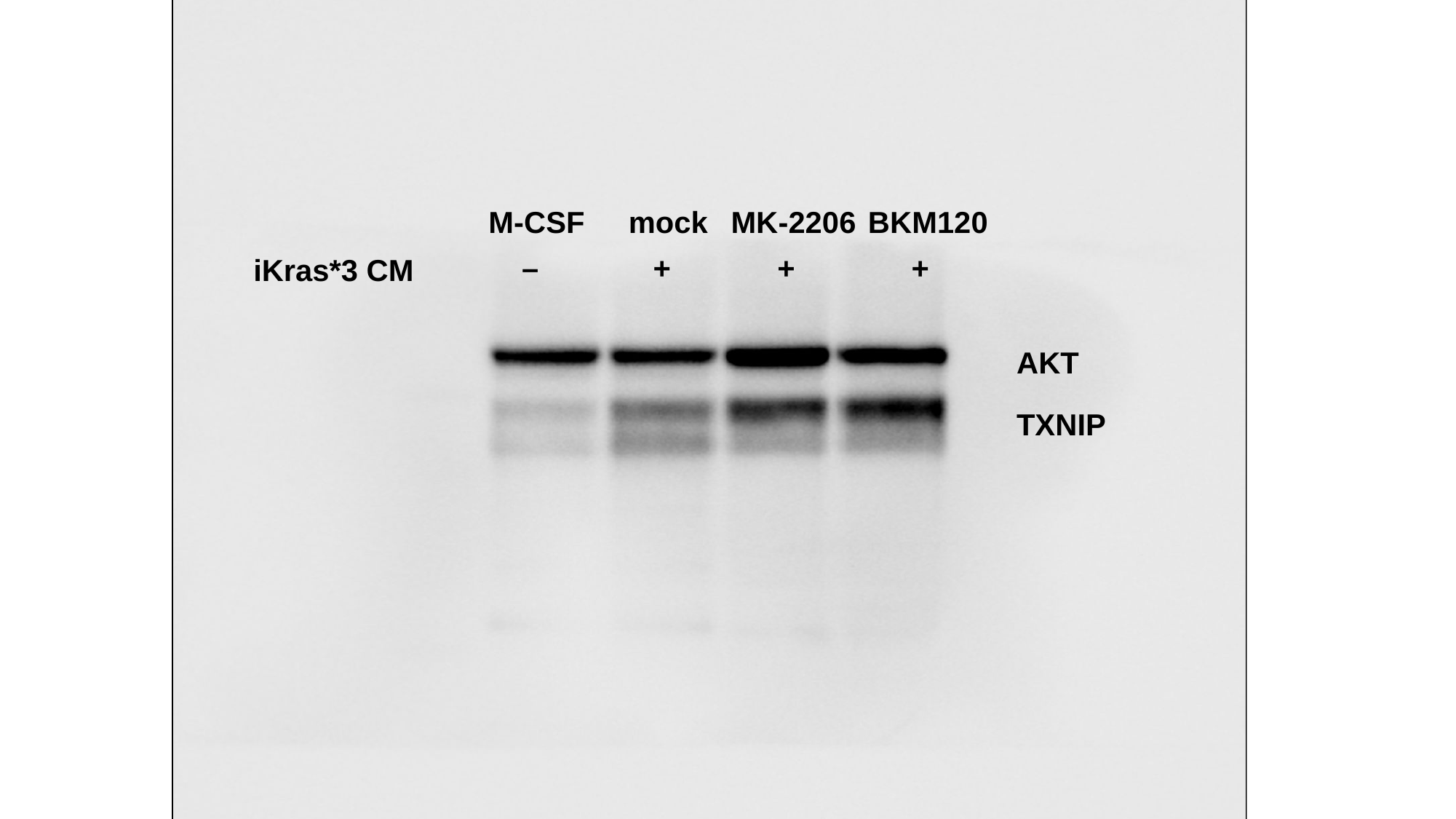

M-CSF
mock
MK-2206
BKM120
–
+
+
+
iKras*3 CM
AKT
TXNIP

## Slide 7
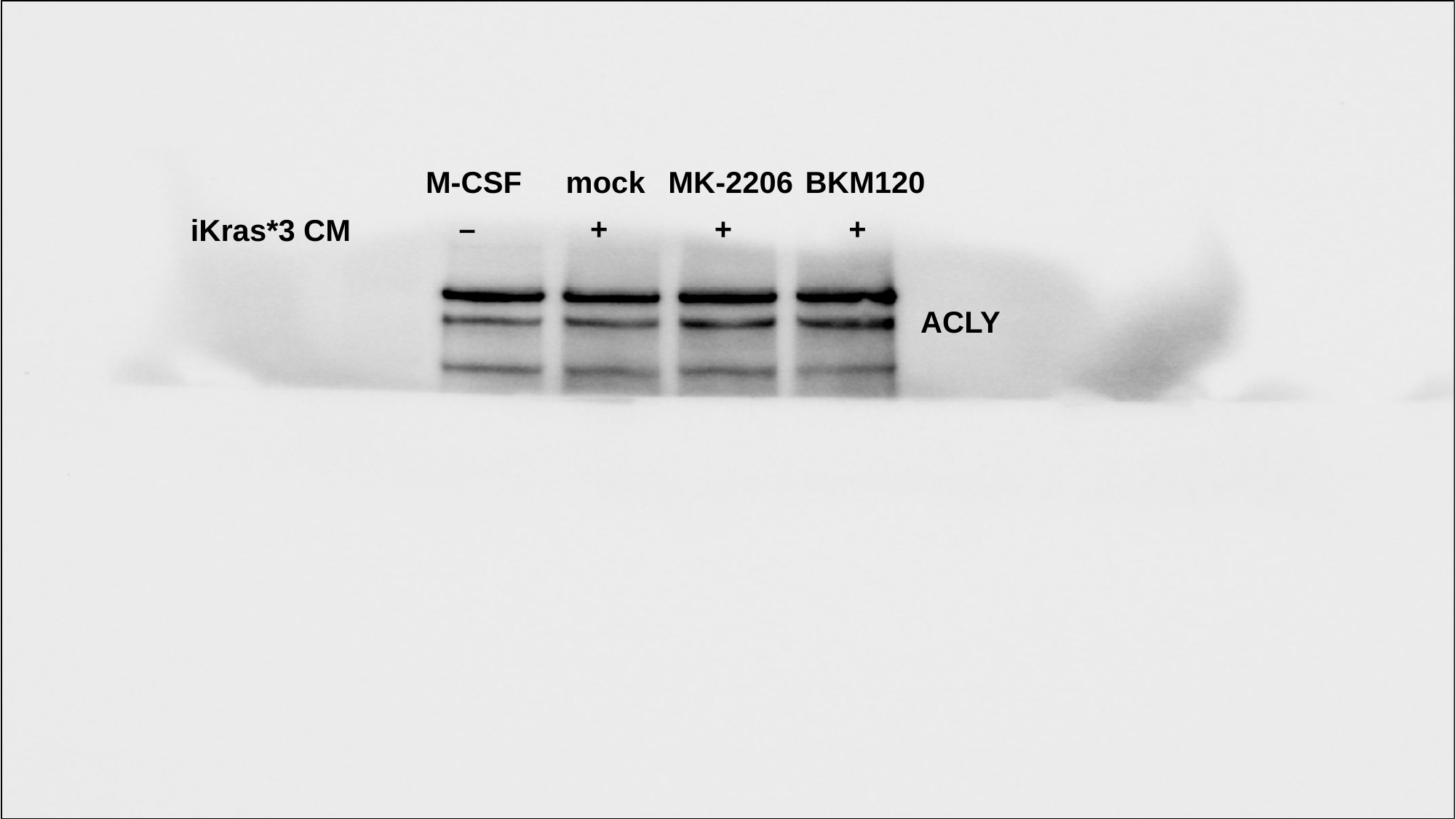

M-CSF
mock
MK-2206
BKM120
–
+
+
+
iKras*3 CM
ACLY

## Slide 8
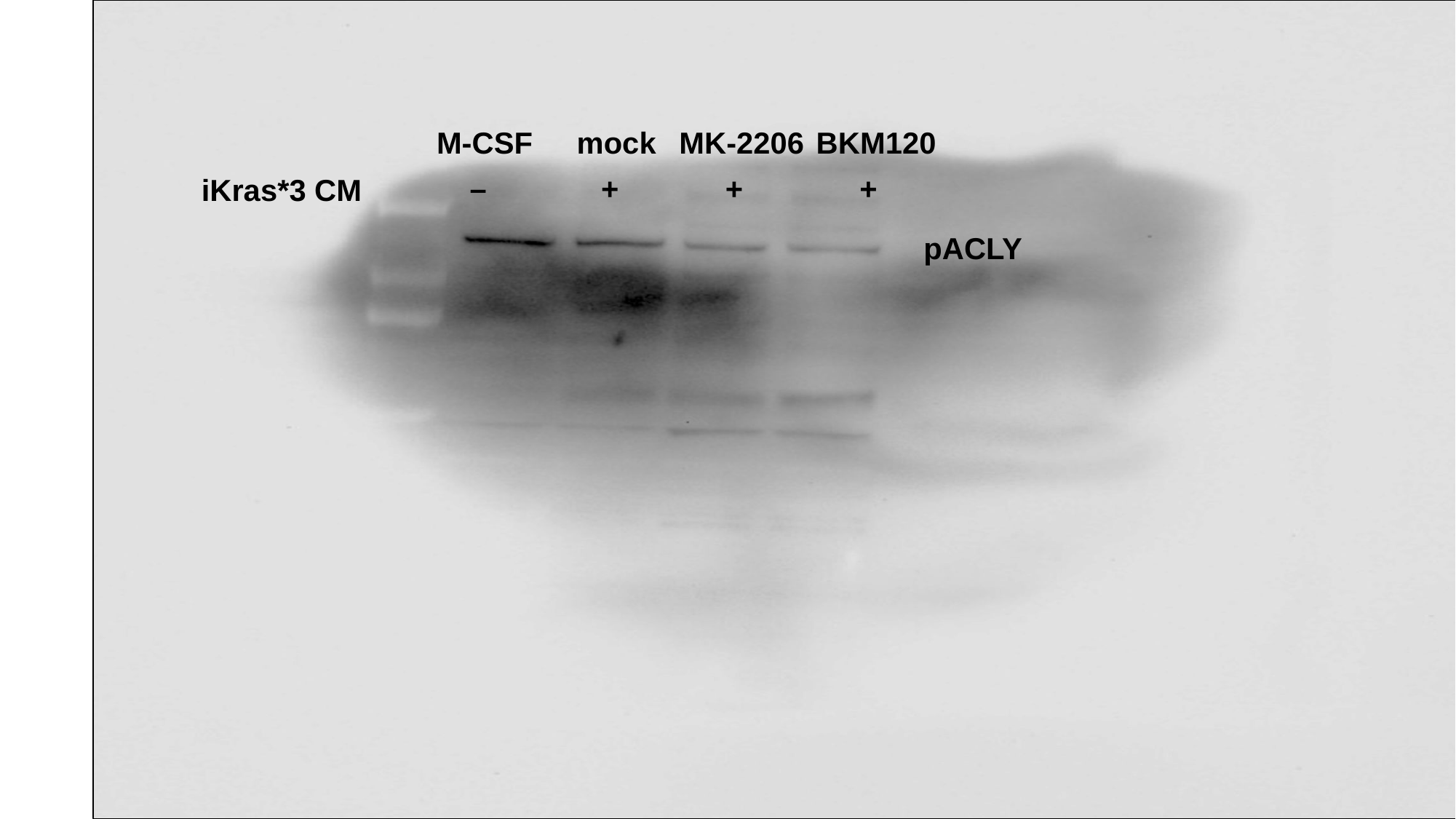

M-CSF
mock
MK-2206
BKM120
–
+
+
+
iKras*3 CM
pACLY

## Slide 9
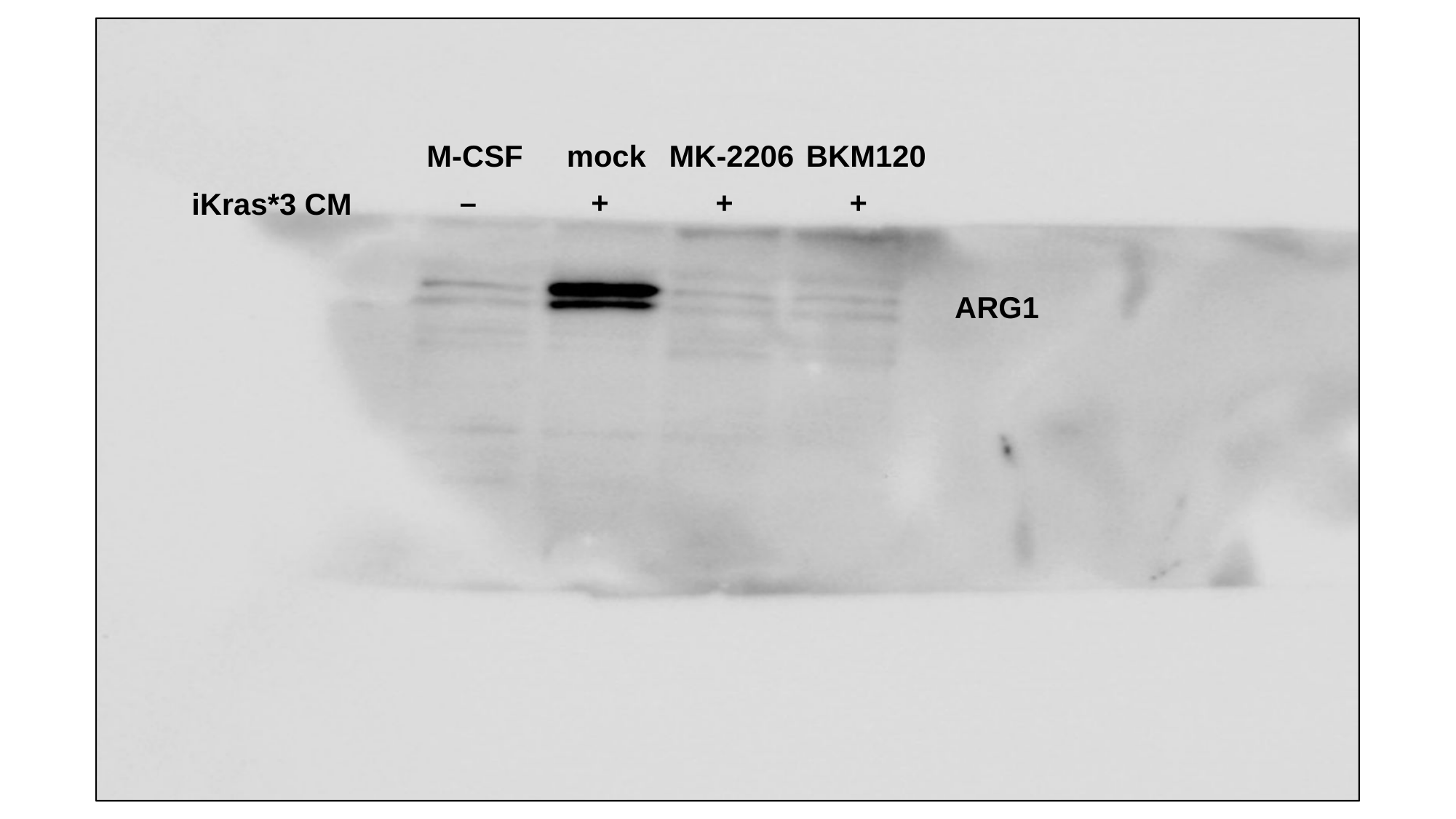

M-CSF
mock
MK-2206
BKM120
–
+
+
+
iKras*3 CM
ARG1

## Slide 10
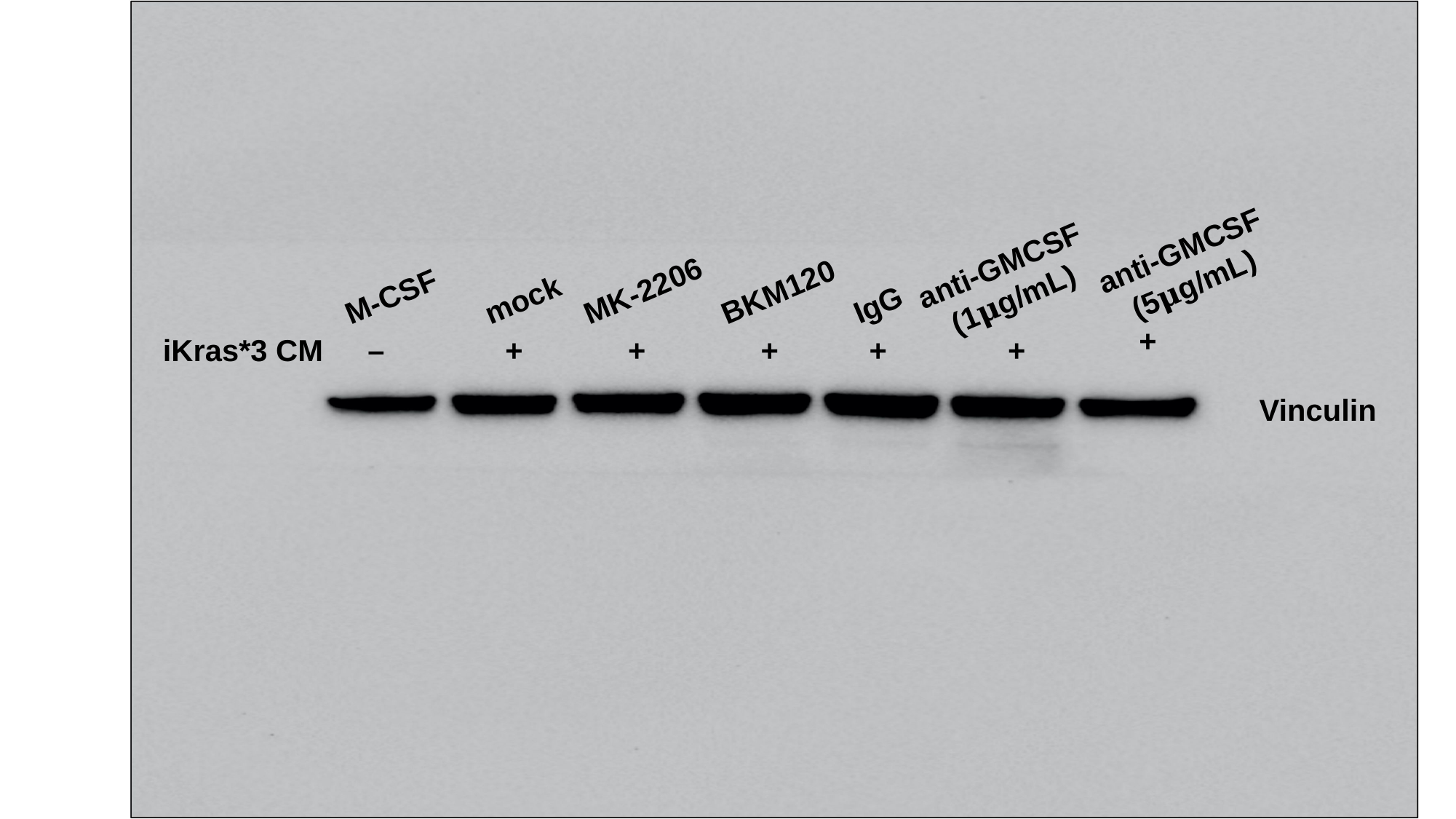

anti-GMCSF (5𝛍g/mL)
anti-GMCSF (1𝛍g/mL)
MK-2206
BKM120
M-CSF
mock
IgG
+
iKras*3 CM
–
+
+
+
+
+
Vinculin

## Slide 11
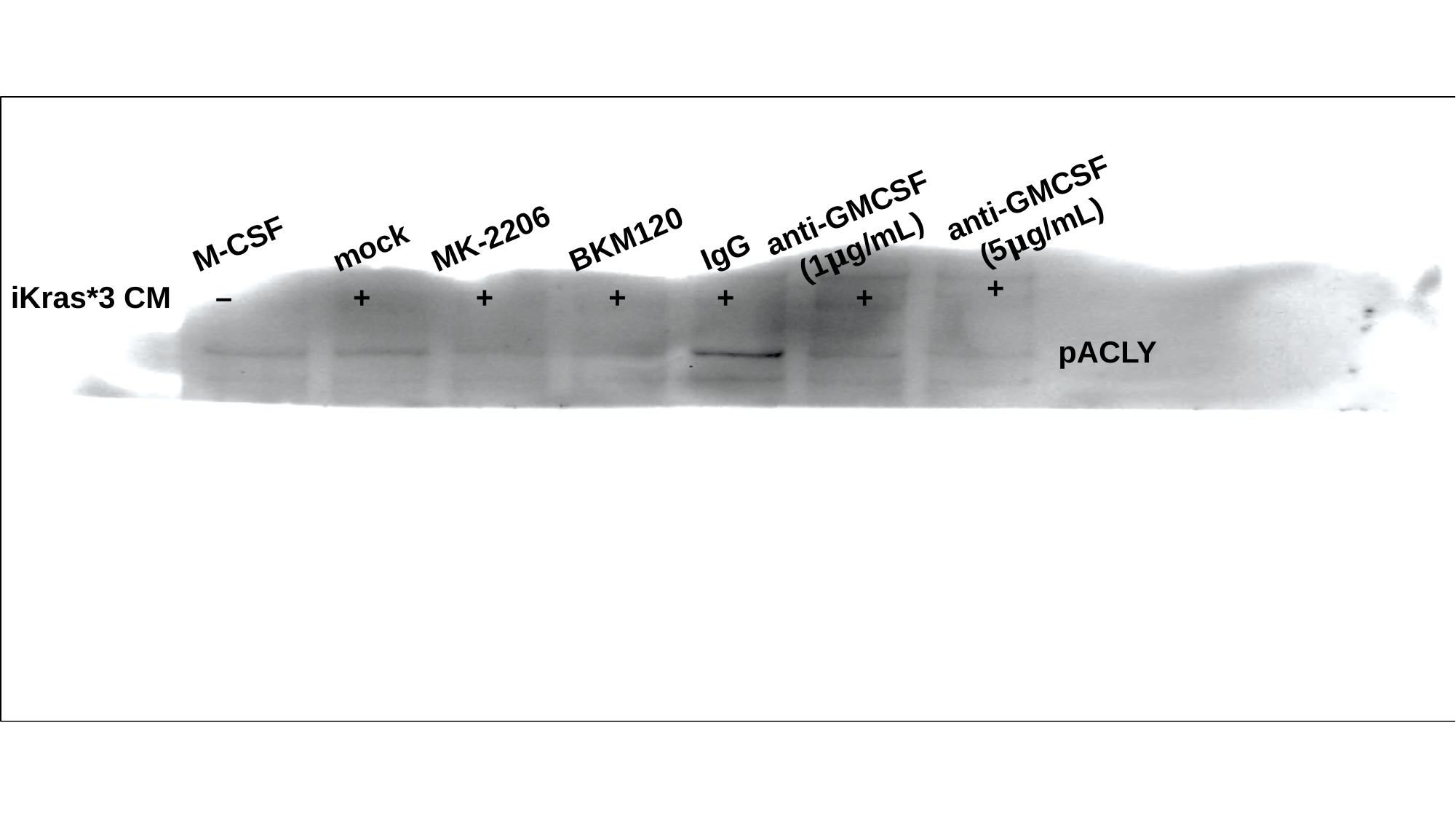

anti-GMCSF (5𝛍g/mL)
anti-GMCSF (1𝛍g/mL)
MK-2206
BKM120
M-CSF
mock
IgG
+
iKras*3 CM
–
+
+
+
+
+
pACLY

## Slide 12
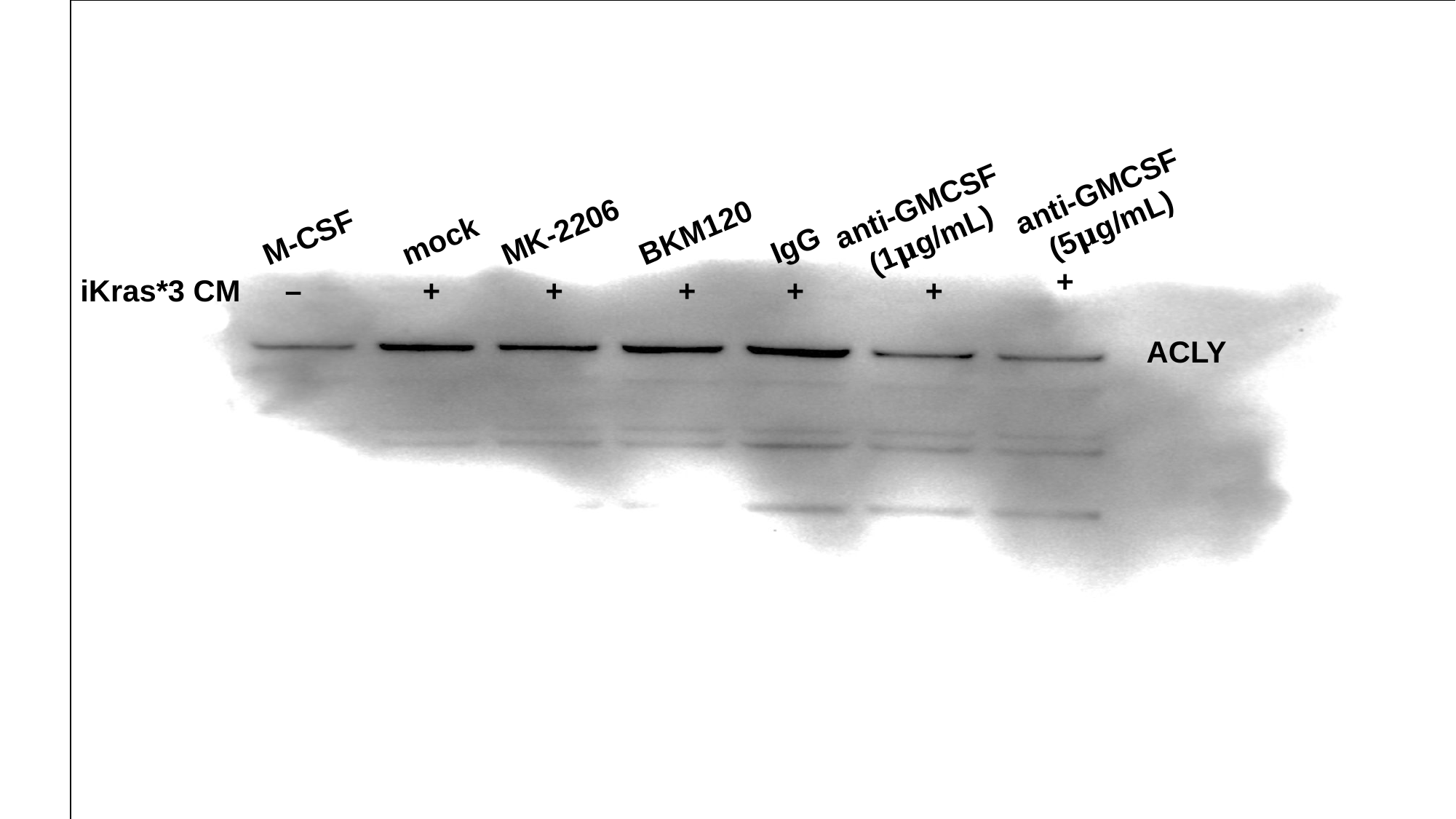

anti-GMCSF (5𝛍g/mL)
anti-GMCSF (1𝛍g/mL)
MK-2206
BKM120
M-CSF
mock
IgG
+
iKras*3 CM
–
+
+
+
+
+
ACLY

## Slide 13
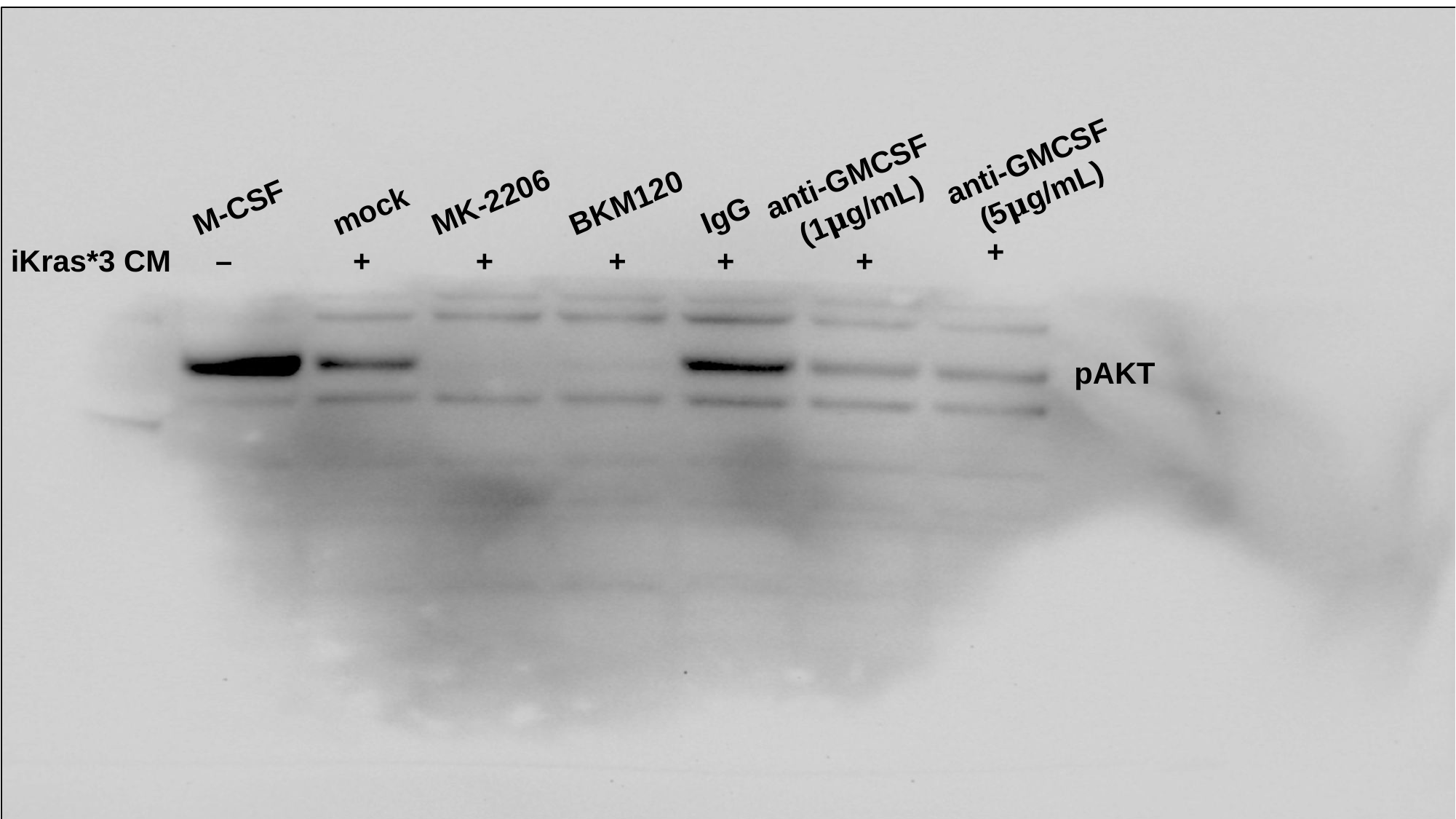

anti-GMCSF (5𝛍g/mL)
anti-GMCSF (1𝛍g/mL)
MK-2206
BKM120
M-CSF
mock
IgG
+
iKras*3 CM
–
+
+
+
+
+
pAKT

## Slide 14
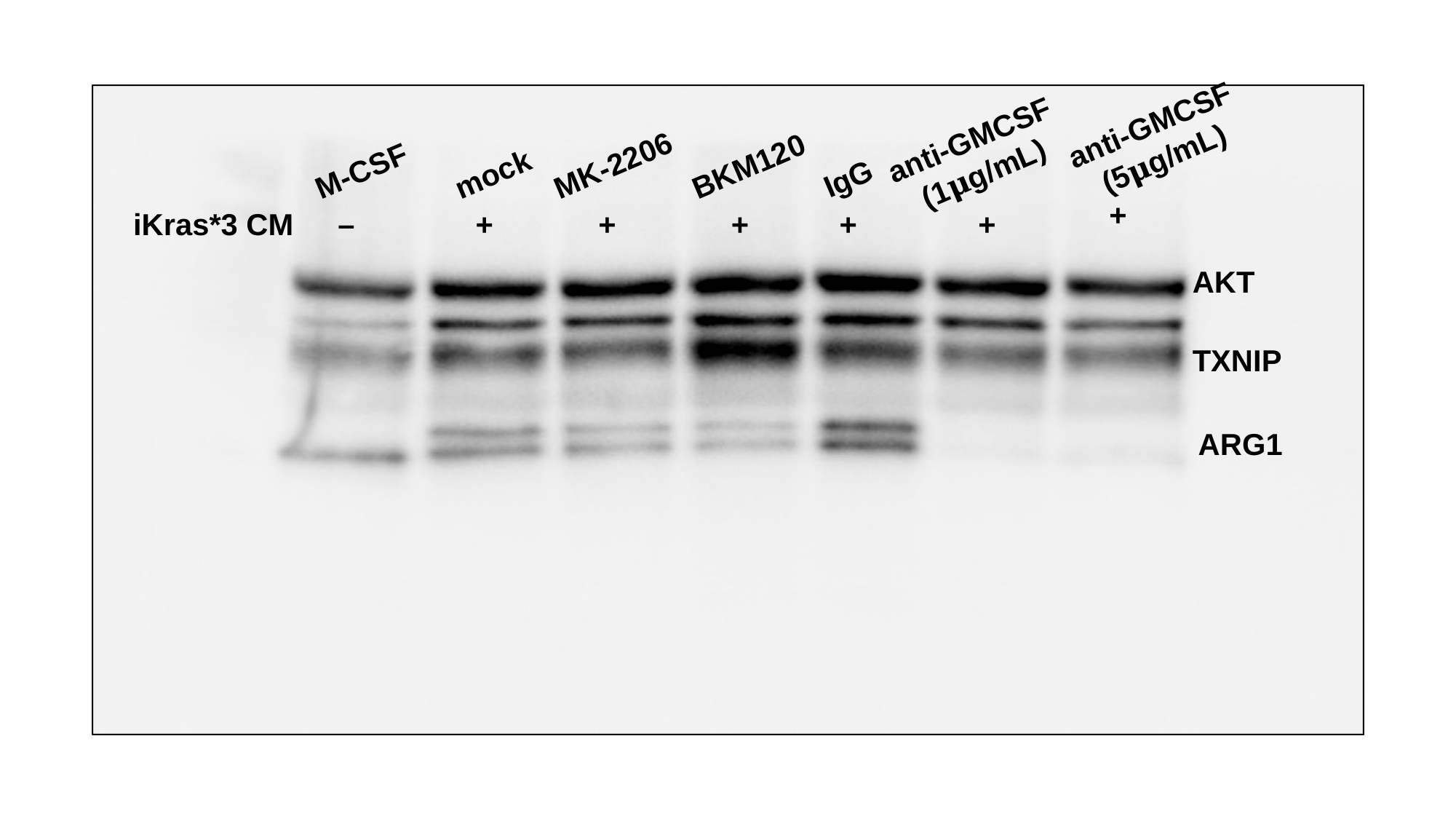

anti-GMCSF (5𝛍g/mL)
anti-GMCSF (1𝛍g/mL)
MK-2206
BKM120
M-CSF
mock
IgG
+
iKras*3 CM
–
+
+
+
+
+
AKT
TXNIP
ARG1

## Slide 15
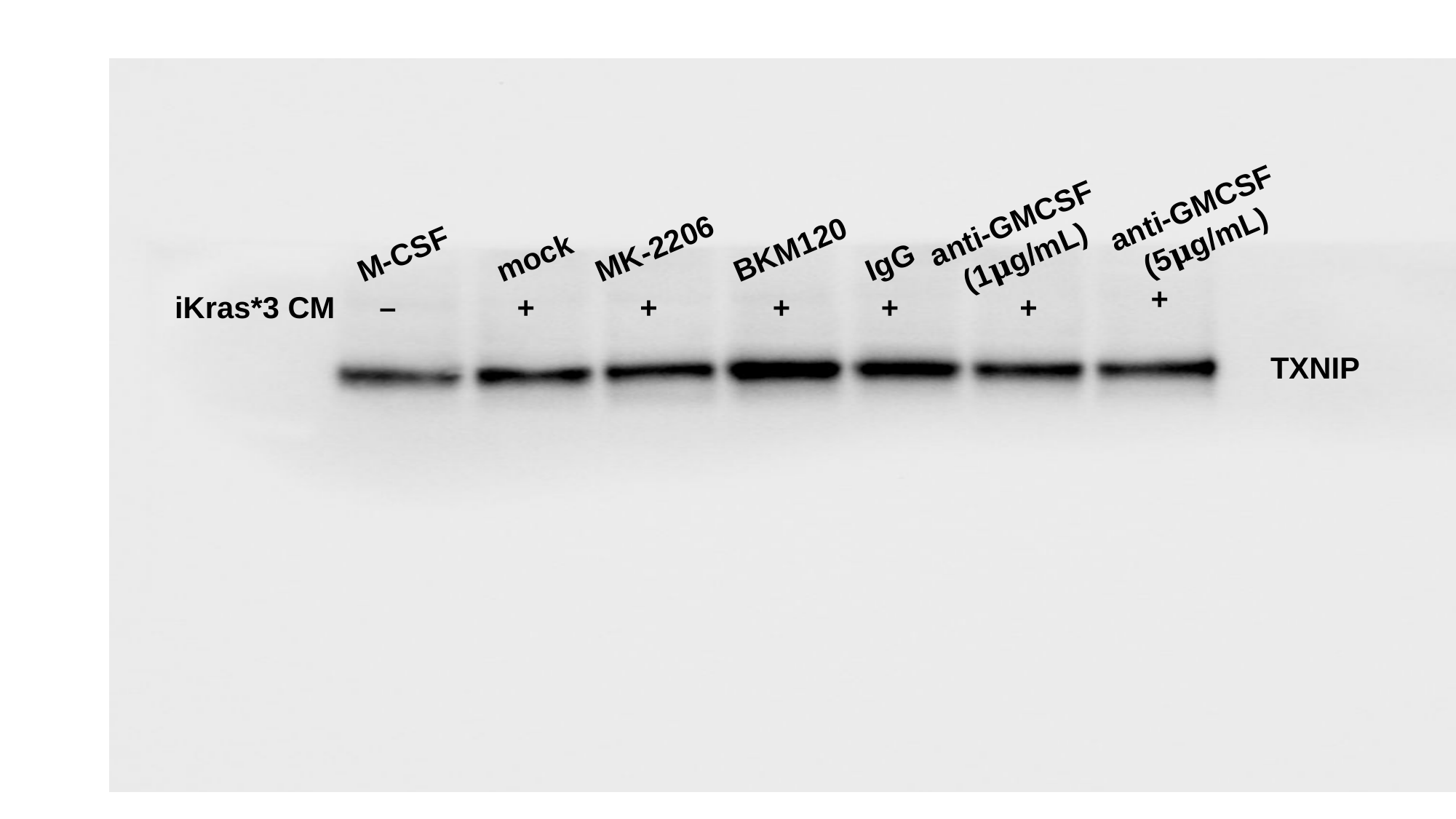

anti-GMCSF (5𝛍g/mL)
anti-GMCSF (1𝛍g/mL)
MK-2206
BKM120
M-CSF
mock
IgG
+
iKras*3 CM
–
+
+
+
+
+
TXNIP

## Slide 16
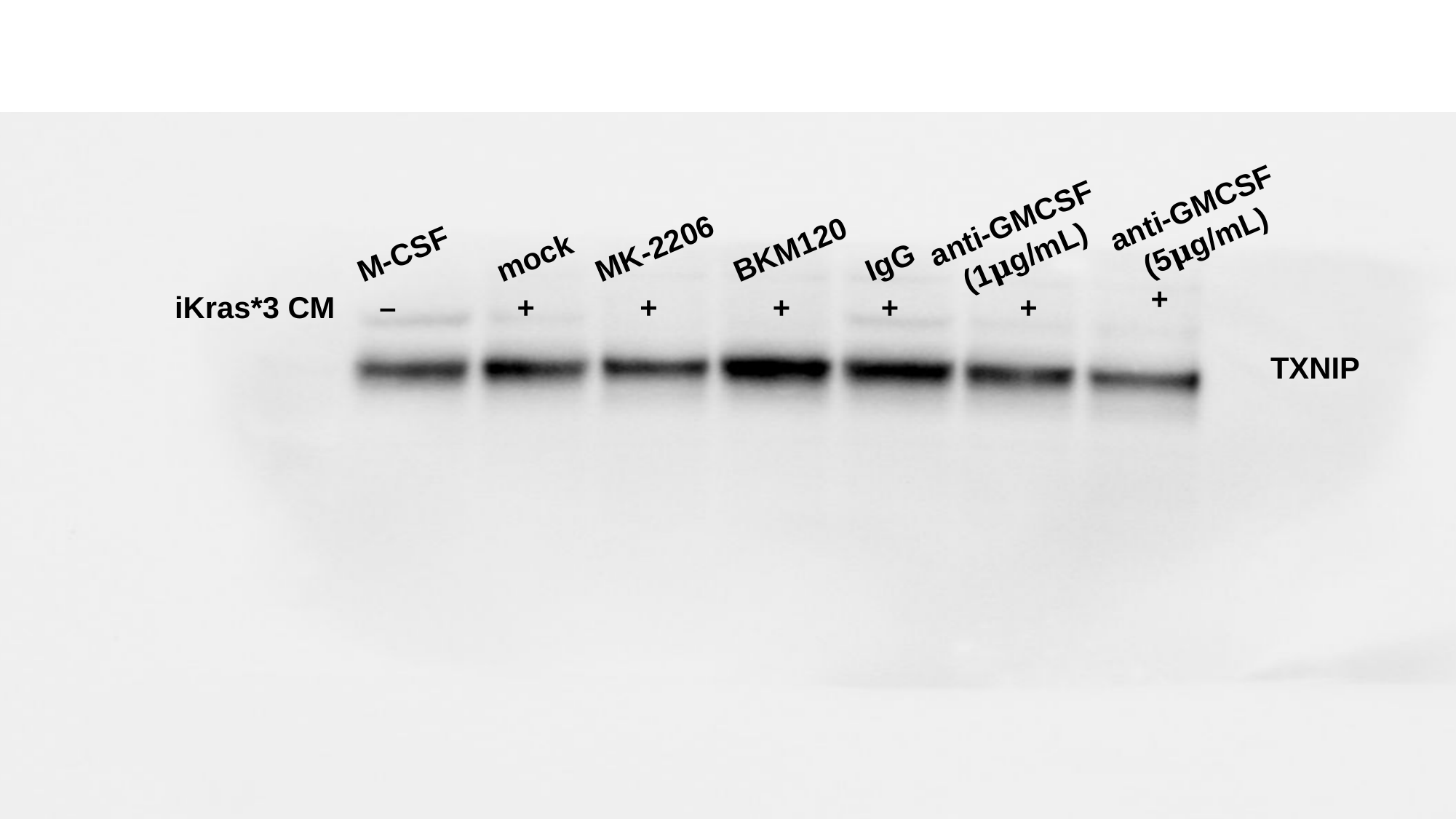

anti-GMCSF (5𝛍g/mL)
anti-GMCSF (1𝛍g/mL)
MK-2206
BKM120
M-CSF
mock
IgG
+
iKras*3 CM
–
+
+
+
+
+
TXNIP
